# Unveiling Maternal Health Insights During the COVID-19 Pandemic in Pakistan: Using Causal Loop Diagrams to Illuminate and Prevent Unintended Policy Effects

**DOI:** 10.9745/GHSP-D-21-00803

**Published:** 2023-10-30

**Authors:** Shamsa Zafar, Pei Shan Loo, Ayesha Rasheeda Avais, Saera Afghan, Don de Savigny, Daniel Cobos Muñoz, Carmen Sant Fruchtman

**Affiliations:** a Fazaia Medical College, Air University, Islamabad, Pakistan.; b Swiss Tropical and Public Health Institute, Basel, Switzerland.; c University of Basel, Basel, Switzerland.; d PAF Hospital Islamabad, Islamabad, Pakistan.; e Pakistan Institute of Medical Sciences, Islamabad, Pakistan.

## Abstract

The authors explain that using causal loop diagrams can visualize retrospectively the unintended negative consequences of COVID-19 related policies on maternal health and has potential to be used prospectively to foster decision-making to prevent those consequences.

## Key Messages

- Causal loop diagrams can highlight and visualize complex behaviors, pinpointing vital leverage points for effective interventions in response to public health crises. Additionally, their use promotes a mutual understanding among policymakers, managers, and service providers about the depth of the crisis and the resulting response.
- We used a causal loop diagram to visualize how the COVID-19 pandemic and its response disrupted maternal health services in Islamabad, Pakistan.
- We argue that given the complexity of the response to the COVID-19 pandemic and the pre-existing vulnerabilities of Pakistan's maternal health system, systems thinking approaches, such as causal loop diagrams, should have been at the center of the pandemic preparedness and response to consider and prevent unintended consequences.

## IMPACT OF COVID-19 ON MATERNAL HEALTH SERVICES IN ISLAMABAD, PAKISTAN

On February 26, 2020, Pakistan reported its first travel-associated confirmed case of COVID-19 in Islamabad. Shortly after, on March 23, 2020, the Government of Pakistan declared a nationwide lockdown, encompassing Islamabad and Rawalpindi. The absence of full-fledged isolation centers in Islamabad led to the implementation of a home-based isolation strategy, accompanied by a 2-week cordoning of the entire area.^1^ The suspension of outpatient services by provincial governments worsened the already precarious access to health services, necessitating the intervention of the Supreme Court of Pakistan to mandate the resumption of these services across all hospitals.^2^

Even before the emergence of the COVID-19 pandemic, Pakistan's health system grappled with barriers that disproportionately impacted marginalized groups and women. These challenges were fueled by inadequate quality of care and a dearth of essential commodities and services.^3^ Notably, the maternal mortality ratio remained alarmingly high, surpassing regional benchmarks.^4^ The structure of Pakistani society is characterized by 2 main institutionalized systems of inequality: class and gender. As a result, even during “normal” times, educated women are over 3 times more likely to be attended by skilled birth personnel than women with no education.^5^

Regrettably, the COVID-19 crisis exacerbated the deterioration of existing health indicators, particularly in the domain of maternal health. The Ministry of Health of Pakistan estimated a reduction of 19% in facility-led births during 2020 due to the impact of the COVID-19 pandemic on health care access and quality.^6^ A study in Pakistan also observed a 37% decrease in institutional deliveries and first postnatal visits.^7^ Behind these statistics were many intertwined reasons that stem from the economic and social effects and the toll that COVID-19 has had on health workers, as well as the measures put in place to stop the spread of the disease.^7^

## DEVELOPMENT OF A CAUSAL LOOP DIAGRAM TO VISUALIZE UNINTENDED MATERNAL HEALTH CONSEQUENCES OF THE COVID-19 PANDEMIC

In this commentary, we use a causal loop diagram (CLD)—a systems thinking tool—to illuminate the complex interplay of factors that collectively contributed to the erosion of access and quality in maternal health services across Islamabad during the first months of the pandemic in 2020.^8^ CLD is a qualitative method used to visually represent the dynamics within a system or scenario, highlighting the intricate interactions among its components.^9^ Within this method, the system's variables are denoted and linked through arrows bearing polarity indicators (+/−), which serve to depict the nature of their relationships. These intervariable relationships can give rise to feedback loops, which are cyclic processes of change. The loops can either augment an effect (resulting in a reinforcing loop [R] that triggers amplification) or counteract it (yielding a balancing loop [B] that promotes stability).^8^

We use a causal loop diagram to illuminate the complex interplay of contributors to the erosion of access and quality in maternal health services across Islamabad early in the pandemic.

We aim to showcase how CLDs, if applied at the right time, can serve as a tool for health managers to avert the adverse outcomes arising from isolated interventions within the realm of public health.^10^ In the subsequent sections, we describe the process through which we formulated the CLD, delineate the principal factors we pinpointed as catalysts for unintended consequences, and describe the potential benefits the CLD could have conferred if used by Ministry of Health officials, district health managers, or hospital administrators.

To develop the CLD, the authors—a group of international researchers, managers, and service providers from the Pakistani health system—held discussions on maternal health care and the impact of COVID-19. The 3 authors from Islamabad had work experience in the maternal health system and used this to orientate the development of the CLD. The resulting diagram, shown in the Figure, was validated with emerging literature on the topic of unintended consequences on the health system (discussed later in the article) when available from Pakistan. The CLD was developed with Stella Architect software.

**FIGURE.**
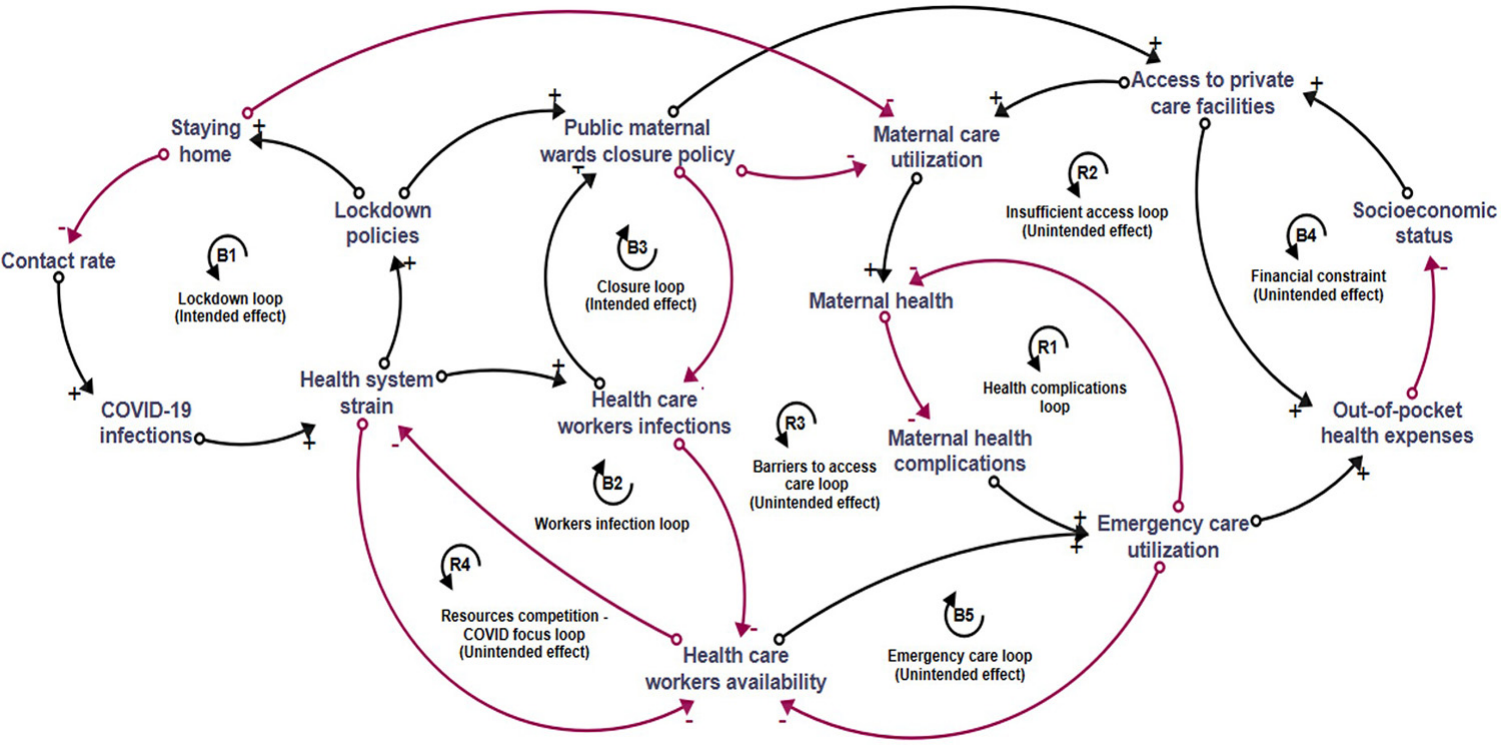
Causal Loop Diagram Depicting the Unintended Effects of the COVID-19 Pandemic and Its Response on Maternal Health Services in Islamabad, Pakistan^a^ ^a^The diagram depicts 5 balancing (B) loops—lockdown, workers infections, health facility closure, financial constraint, and emergency care—that stabilize the effect of the COVID-19 pandemic and depicts 4 reinforcing (R) loops—health complications, insufficient access, barriers to access care, and resources competition—that amplify the effect. Arrows represent the direction of effect between variables: red indicates negative (−) relationships and black indicates positive (+) relationships.

## CONSEQUENCES OF THE COVID-19 PANDEMIC AND RESPONSE POLICIES ON MATERNAL HEALTH

During the first months of the pandemic in 2020, the surge in COVID-19 cases, coupled with infections among health professionals, rapidly overwhelmed public health facilities.^11^ The implementation of lockdown policies aimed to encourage people to stay at home, effectively reducing the contact rate and subsequently decreasing COVID-19 infections, as illustrated in the lockdown loop (B1) in the Figure. This intended effect ultimately led to a decline in health care utilization, thereby contributing to a reduction in the infection rate among health care workers, as shown in the workers' infection loop (B2). In addition, the closure loop (B3) illustrates an attempt by the government to prevent further infections among health personnel and prioritize resources for COVID-19 by closing outpatient departments from public sector hospitals.

The inclusion of the maternal wards' closure policy within the lockdown measures resulted in a number of unintended consequences, surpassing the intended reduction in hospital transmissions. Many pregnant women were left with the only option to seek care in private hospitals. The financial constraint loop (B4) shows that for women of lower socioeconomic status, the financial burden of private services was restrictive, resulting in their exclusion from critical services such as antenatal care and skilled birth attendance.^12^ The health complications loop (R1) shows how the lack of antenatal and postpartum care during the COVID-19 waves resulted in an increase in the number of maternal emergency complications (e.g., eclampsia, uterine scar rupture, and intrauterine deaths).^7^

On the other hand, approximately 85% of all health services were provided by private health facilities even before the pandemic.^13^ Yet, while those who could afford private care had this recourse, the available services often fell short of optimal quality. These private hospitals were not always equipped to handle high-risk deliveries, creating a situation where access to superior care was unattainable. The dynamics are illustrated in the insufficient access loop (R2).

The lockdown measures also resulted in a series of unintended consequences on the demand side among pregnant women and mothers. A protocol was introduced that required COVID-19 screening before any intervention. This approach excluded COVID-19-positive patients, including pregnant women, from receiving adequate treatment in public facilities.^14^ The barriers to access care loop (B3) demonstrates that the community's fear of testing positive and facing social exclusion (including from health services), as well as the rising social alarm around the danger of getting infected in health facilities, contributed to a reduction and delay in care-seeking.^12^ Delays were also influenced by movement restrictions, such as the requirement of travel permission, which increased travel distances to access available services, as well as reduced household income during the pandemic.^12^ Mothers, facing accumulated health issues from service interruptions during the lockdown, began presenting more frequently with complications (health complications loop, R1). This surge in demand illuminated an unintended consequence of the lockdown: the pressing need for more health care workers in the realm of maternal care, as illustrated by the emergency care loop (B5).

As the pandemic raged on, the demand for health care professionals in maternal care services began to directly compete with the urgent needs of COVID-19-focused responses, as outlined in the resources competition loop (R4). This tug-of-war for health care workers and resources highlighted a crucial imbalance. The very policies aiming to curtail the spread of COVID-19 inadvertently catalyzed more maternal complications, increased the need for emergency care, led to a dwindling availability of health care workers, and exerted greater strain on an already stretched health system.

Policies to curtail the spread of COVID-19 inadvertently catalyzed more maternal complications and exerted greater strain on an already stretched health system.

Overall, lockdown policies, implemented with the primary intention of curbing the spread of COVID-19, had inadvertently resulted in a series of unintended consequences on maternal health. While these policies were critical in managing the immediate threat of the pandemic, the subsequent disruptions in maternal care services became a significant collateral issue. The unintended impacts on maternal health serve as a poignant reminder of the delicate balance required in health system responses during global crises.

## STRENGTHS AND LIMITATIONS OF APPLYING A CLD TO SUPPORT PUBLIC HEALTH RESPONSE DURING CRISES

The application of a CLD stands as a promising means to bolster the capacities of decision-makers in preparing for forthcoming crises. In the example previously mentioned, we have visualized retrospectively how the lockdown and ward closure policies led to unintended consequences, including severe effects on maternal health. However, the real potential of a CLD lies in its prospective use, which, when combined with real-time surveillance data, can be crucial.

This tool has the potential to serve as a dynamic framework that fosters proactive strategies through enhanced stakeholder collaboration.^9^ By facilitating robust exchanges among various stakeholders, the CLD becomes a conduit for surfacing insights that are pivotal in recognizing potential unintended consequences, such as those that occurred on maternal and newborn health during the COVID-19 pandemic in Pakistan.^15^ Over the past 2 decades, CLDs have gained momentum as a potent mechanism for facilitating discussions and aiding decision-making among stakeholders spanning various domains. This emphasizes the notion that applying the tool on the cusp of the COVID-19 outbreak—not merely as a research instrument but as a pragmatic decision-making aid—could have been pivotal.

Of course, such a proposition would necessitate that decision-makers possess a literacy in the language of CLDs or collaborate with those that do (e.g., researchers), along with an understanding of the participatory approaches that render CLDs effective. The authors' engagement with district health managers in Pakistan during the pandemic was geared toward this very objective. In our experience, which we described elsewhere,^16^ the utility of systems thinking tools—in that instance, process mapping—proved useful in casting a comprehensive, system-wide perspective on COVID-19 contact tracing.

Although the benefits of employing CLDs in crisis response are evident, the practical implementation may encounter hurdles when attempting to engage all stakeholders and facilitate in-depth discussions. Balancing the need for comprehensive insights with the exigencies of timely decision-making remains a delicate challenge, underscoring the nuanced interplay between the potential strengths and limitations of this approach.

### Implications for Future Emergencies

The aftermath of COVID-19 has generated unprecedented challenges while unmasking the vulnerabilities inherent within maternal health care systems worldwide.^17^ Although the response to COVID-19 required social-distancing measures, as well as the redesign of hospital systems and staff, it cannot come at the expense of closing or denying care for already existing conditions, such as maternal and neonatal emergencies that will occur regardless of the pandemic.

When designing public health interventions and policies specifically for maternal health, and especially within the response to pandemics, decision-makers must consider the different needs of populations, as well as how single interventions can have effects on other seemingly “disconnected” areas. Had policymakers foreseen these unintended consequences, they might have designed policies that prevent disruption to maternal care. Hence, we advocate for the integration of systems thinking approaches in shaping interventions and policies, not only as a response to past crises like COVID-19 but also as a foundation for enhancing emergency preparedness and response to future outbreaks.^18^ Early use of CLDs with the participation of health workers could have illuminated these unintended effects and potential mitigation options earlier in the policy process.

Finally, embracing a holistic understanding of the health system and beyond must be accompanied by acknowledging and addressing the varied experiences of individuals. COVID-19 is reinforcing and multiplying existing structures of discrimination, and, at the same time, it is showing that no one will be safe until all are.^19^ It is the government's responsibility to formulate holistic policy strategies that anticipate possible behavioral outcomes and embed safeguards to avoid unintended negative impacts. Hence, in embracing a systemic approach, it is paramount to center the distinctive needs and challenges encountered by pregnant individuals while acknowledging the intricate intersections that shape their experiences.^19^

## Notes

**Peer Reviewed**

**First published online:** October 17, 2023.
